# MCU (mitochondrial Ca^2+^ uniporter) makes the calcium go round

**DOI:** 10.1016/j.jbc.2022.101604

**Published:** 2022-01-17

**Authors:** Grant C. Walters, Yuriy M. Usachev

**Affiliations:** Department of Neuroscience and Pharmacology, Iowa Neuroscience Institute, University of Iowa, Iowa City, Iowa, USA

**Keywords:** calcium, CRAC channels, MCU, mitochondria, NFAT, SOCE, lymphocyte, [Ca^2+^]_cyt_, cytosolic Ca^2+^ concentration, CDI, Ca^2+^-dependent inactivation, CRAC, Ca^2+^ release–activated Ca^2+^, ER, endoplasmic reticulum, IL, interleukin, IP_3_, inositol-1,4,5-trisphosphate, MCU, mitochondrial Ca^2+^ uniporter, NFAT, nuclear factor of activated T cells, SOCE, store-operated Ca^2+^ entry

## Abstract

Store-operated Ca^2+^ entry (SOCE) is a major mechanism controlling Ca^2+^ signaling and Ca^2+^-dependent functions and has been implicated in immunity, cancer, and organ development. SOCE-dependent cytosolic Ca^2+^ signals are affected by mitochondrial Ca^2+^ transport through several competing mechanisms. However, how these mechanisms interact in shaping Ca^2+^ dynamics and regulating Ca^2+^-dependent functions remains unclear. In a recent issue, Yoast *et al.* shed light on these questions by defining multiple roles of the mitochondrial Ca^2+^ uniporter in regulating SOCE, Ca^2+^ dynamics, transcription, and lymphocyte activation.

Calcium signaling regulates many fundamental cell functions, including gene expression, exocytosis, motility, and proliferation. A common mechanism for generating such signals involves Ca^2+^ release from the endoplasmic reticulum (ER) in response to the generation of the inositol-1,4,5-trisphosphate (IP_3_) second messenger following cell stimulation by various hormones and growth factors. Depletion of ER Ca^2+^ triggers the activation of Ca^2+^ release–activated Ca^2+^ (CRAC) channels in the plasma membrane allowing Ca^2+^ influx into the cell (1, 2). This fundamental mechanism known as store-operated Ca^2+^ entry (SOCE) serves to refill ER Ca^2+^ stores, shape cytosolic Ca^2+^ signaling, and regulate numerous Ca^2+^-dependent functions. Once activated, CRAC channels undergo Ca^2+^-dependent inactivation (CDI), which limits SOCE through a negative feedback mechanism (1, 2). Mitochondria localized close to the CRAC channels prevent CDI by buffering Ca^2+^ *via* the mitochondrial Ca^2+^ uptake complex (3, 4), which in theory should amplify CRAC-mediated increase in cytosolic Ca^2+^ concentration ([Ca^2+^]_cyt_). However, the net effect of mitochondria on the Ca^2+^ signal depends on several additional factors, including the ability of mitochondria to release accumulated Ca^2+^ back into the cytosol, regulate Ca^2+^ dynamics within the ER, and generate ATP in a Ca^2+^-dependent manner. The complex problem of how these mitochondria-dependent mechanisms work together to shape SOCE-mediated Ca^2+^ signaling is the focus of the study by Yoast *et al.* (5).

Compared with earlier work that relied on pharmacological tools of limited specificity, Yoast *et al.* (5) employed new molecular tools, which were not available at the time when mitochondria-dependent regulation of CRAC channels was discovered more than 20 years ago (6, 7). They used CRISPR–Cas9 to delete the core molecular component of the mitochondrial Ca^2+^ uptake complex, mitochondrial Ca^2+^ uniporter (MCU) (3, 4), in various cell lines from different tissues and species and examined the effects of MCU KO on SOCE-mediated [Ca^2+^]_cyt_ changes (5). Unexpectedly, and in contrast to the postulated role of mitochondrial Ca^2+^ buffering in supporting SOCE, they found that MCU KO led to an increase, rather than a decrease, in SOCE-mediated [Ca^2+^]_cyt_ transients (5). A similar increase was observed in native T and B cells from conditional MCU KO mice.

The authors then systematically examined the effects of MCU KO on other mechanisms contributing to Ca^2+^ signaling. First, using whole-cell patch-clamp recordings, they found that MCU KO promoted inactivation of CRAC currents, consistent with previous reports that mitochondrial Ca^2+^ buffering reduces CDI of CRAC channels (6, 7). Second, using subcellular Ca^2+^ imaging, they showed that MCU KO led to accelerated refilling of ER Ca^2+^ stores and increased ER Ca^2+^ content under resting conditions but did not alter activity of the IP_3_ receptors (5). Third, dissipation of the mitochondrial electrochemical gradient with a protonophore, carbonylcyanide p-trifluoromethoxyphenylhydrazone, blocked SOCE in both WT and MCU KO cells (5), suggesting that the carbonylcyanide p-trifluoromethoxyphenylhydrazone effect was independent of mitochondrial Ca2^+^ uptake. This finding is particularly insightful because it helps to explain discrepancies between earlier works that relied on the use of protonophores (*e.g.*, carbonyl cyanide *m*-chlorophenyl hydrazone) and electron transport inhibitors (*e.g.*, antimycin A) to block mitochondrial Ca^2+^ buffering by inducing mitochondrial depolarization and thereby dissipating the driving force for mitochondrial Ca^2+^ uptake (6, 8). Although both carbonyl cyanide *m*-chlorophenyl hydrazone and antimycin A blocked SOCE-mediated [Ca^2+^]_cyt_ increase in those studies (6, 8), Yoast *et al.* (5) now clarify that those effects were independent of mitochondrial Ca^2+^ uptake and likely caused by disrupted mitochondrial respiration.

This study also examined the functional significance of MCU-dependent regulation of SOCE-induced Ca^2+^ signaling (5). The authors focused on the Ca^2+^/calcineurin-dependent transcription factor NFAT (nuclear factor of activated T cells) and its control of immune function (9). This choice was well justified, given the central role of SOCE in the regulation of NFAT and NFAT-dependent control of the expression of interleukin 2 (IL-2), IL-4, IL-10, and other cytokines critical for T-cell and B-cell activation and proliferation (1, 2, 10). First, by monitoring SOCE-induced nuclear translocation of NFAT, Yoast *et al.* showed that MCU KO significantly facilitated activation and nuclear import of NFAT. Second, using conditional MCU KO mice, they found that MCU knockdown specifically in B cells significantly enhanced proliferation of these cells in response to B-cell receptor stimulation. Based on these experiments, the authors concluded that MCU KO/knockdown facilitates NFAT activation and lymphocyte proliferation, consistent with the enhancement of SOCE-driven [Ca^2+^]_cyt_ elevations in MCU KO cells (5) (Fig. 1). Notably, these findings challenge the conclusion from earlier work that blocking mitochondrial Ca^2+^ uptake diminishes NFAT activation in immune cells (6). It is important to note that this earlier conclusion was based on the use of a protonophore to block mitochondrial Ca^2+^ uptake; as now demonstrated by Yoast *et al.*, protonophores block SOCE independent of MCU, explaining the difference between this work (5) and earlier findings (6).

**Figure 1:**
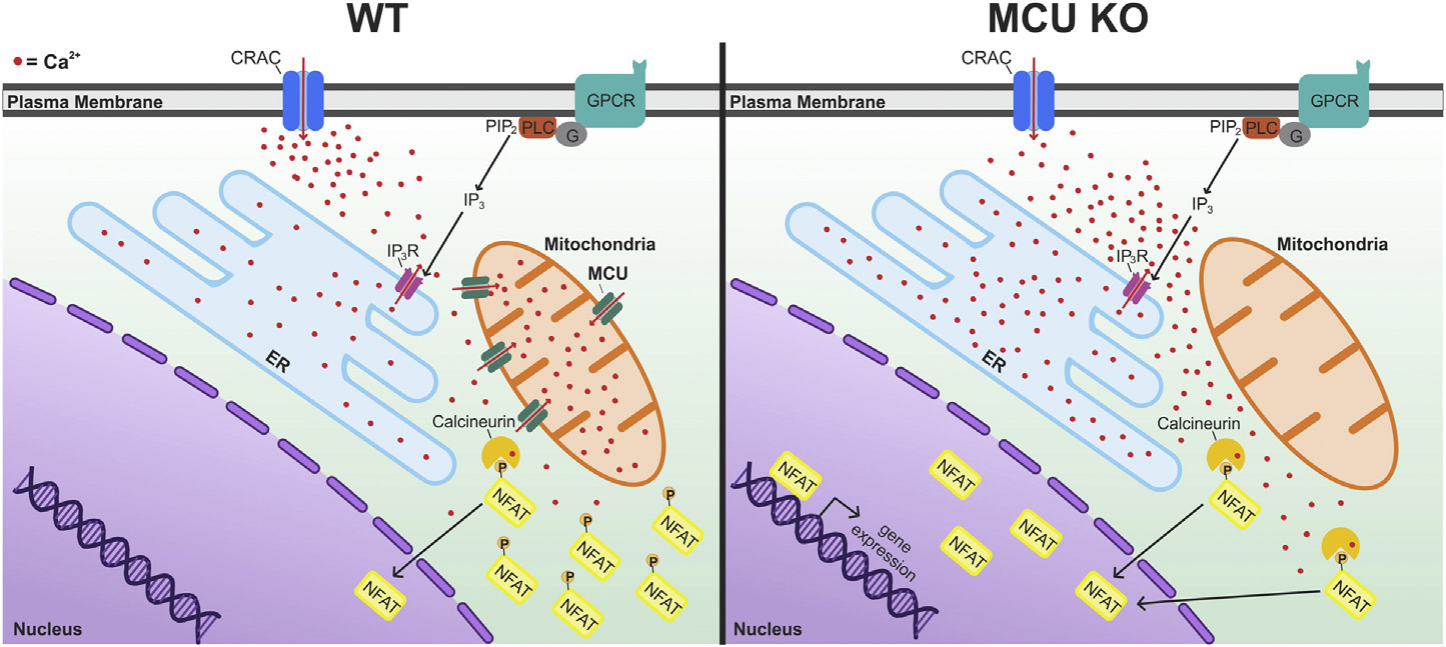
**Summary of the effects of MCU deletion on SOCE-induced cytosolic and organellar Ca**^**2+**^**signaling and activation of the Ca**^**2+**^**-dependent transcription factor NFAT.** Activation of G protein–coupled receptors (GPCRs) or tyrosine kinase receptors can initiate phospholipase C (PLC)-dependent synthesis of the lipid messenger IP_3_ that triggers IP_3_-receptor (IP_3_R)-mediated Ca^2+^ release from the ER. The resulting depletion of the ER Ca^2+^ stores induces SOCE. This contributes to Ca^2+^ (*red dots*) accumulation in the cytosol and activation of the transcription factor NFAT. In WT cells, MCU-mediated Ca^2+^ uptake by mitochondria reduces CDI of CRAC channels, diminishes refilling of the ER stores with Ca^2+^ and limits the global cytosolic Ca^2+^ elevation and activation of NFAT. MCU deletion (MCU KO) reverses all these effects, ultimately resulting in an amplified cytosolic Ca^2+^ elevation, enhanced NFAT activation, and translocation to the nucleus to initiate a transcription response. Please note that the depiction of the mitochondrial Ca^2+^ transport has been simplified for clarity. A detailed description of the MCU complex and of mitochondrial Ca^2+^ efflux systems have been reviewed elsewhere (3, 4). CDI, Ca^2+^-dependent inactivation; CRAC, Ca^2+^ release–activated Ca^2+^; ER, endoplasmic reticulum; IP_3_, inositol-1,4,5-trisphosphate; MCU, mitochondrial Ca^2+^ uniporter; NFAT, nuclear factor of activated T cells; SOCE, store-operated Ca^2+^ entry.

Overall, this study demonstrates that MCU controls multiple aspects of SOCE-mediated Ca^2+^ signaling, including buffering cytosolic Ca^2+^, reducing CDI of CRAC channels, and regulating ER Ca^2+^ store replenishment (Fig. 1). The MCU KO experiments also revealed that the overall contribution of mitochondrial Ca^2+^ buffering predominates among these multiple competing processes. Despite increased CDI and accelerated Ca^2+^ extrusion, the net effect of MCU deletion was an increase, rather than a decrease, in SOCE-mediated [Ca^2+^]_cyt_ transients (Fig. 1). This important conclusion was further supported by extensive mathematical modeling, which systematically tested various contributing factors including CRAC microdomains, CDI, and mitochondrial Na^+^/Ca^2+^ exchange.

As with any insightful study that moves the field forward, the article by Yoast *et al.* outlines new important questions for future research. First, while this work focuses on the role of MCU in shaping cytosolic Ca^2+^ signals, intramitochondrial Ca^2+^ regulates many important processes, including ATP synthesis, oxidative stress, and mitochondrial fission. Hence, an important question is how these multiple MCU-dependent functions act in concert to modulate cellular processes triggered by CRAC activation. Second, it is critical to systematically assess the role of MCU–SOCE interaction in regulating other effectors of Ca^2+^ signaling besides NFAT, including Ca^2+^-dependent enzymes, other transcription factors, cytoskeletal proteins, and Ca^2+^ sensors regulating secretion. Finally, unraveling how MCU regulates SOCE and Ca^2+^-dependent functions in various cell types, including excitable cells (*e.g.*, neurons and muscles) and nonexcitable cells (*e.g.*, platelets, macrophages, and astrocytes), and determining how the cell type–specific molecular composition of MCU complexes and CRAC channels and their subcellular localizations are optimized for the control of distinct functions (*i.e.*, cell migration, muscle contraction, or transmitter release), are important questions to examine. Future research will help address these and many other critical questions inspired by this article.

## Conflict of interest

The authors declare that they have no conflicts of interest with the contents of this article.
